# Modulatory effects of *Lycium barbarum* polysaccharide on bone cell dynamics in osteoporosis

**DOI:** 10.1515/med-2024-1104

**Published:** 2025-02-18

**Authors:** Qing Wang, Ting Zhang, Xinting Feng, Peng Chen, Ye Feng, Haoqiang Huang, Yinhua Qian, Yang Guo, Zifei Yin

**Affiliations:** Department of Orthopaedics, Kunshan Affiliated Hospital of Nanjing University of Chinese Medicine, Kunshan, Jiangsu, 215300, China; Department of Integrative Medicine, Huashan Hospital, Fudan University, Shanghai, China; Department of Sports Medicine, Huashan Hospital, Fudan University, Shanghai, 200040, China; Department of Sports Medicine, Peking University Shenzhen Hospital, Shenzhen, 518000, China; School of Stomatology, Xuzhou Medical University, Xuzhou, Jiangsu, 221000, China; Department of Traumatology and Orthopedics, Affiliated Hospital of Nanjing University of Chinese Medicine, Nanjing, Jiangsu, China; Department of Orthopaedics, Kunshan Affiliated Hospital of Nanjing University of Chinese Medicine, No. 388 Zu Chong Zhi Road, Kunshan, Jiangsu, 215300, China

**Keywords:** osteoporosis, *Lycium barbarum* polysaccharide, osteoblast/osteoclast differentiation, proliferation, apoptosis, signaling pathways

## Abstract

**Background:**

Osteoporosis (OP) is a systemic bone disorder marked by reduced bone mass and disrupted microstructure, leading to higher fracture risk. Epidemiological data from China show a 20.7% prevalence in women and 14.4% in men over 50, underscoring a pressing health issue given the aging population. More drugs to inhibit OP progression should be explored, and their biological mechanisms confirmed in preclinical studies.

**Methods:**

In this study, we utilized *Lycium barbarum* polysaccharide (LBP), an extract from the traditional Chinese medicine Goji Berry. LBP, known for its range of pharmacological activities, was assessed for its potential therapeutic effects on OP. We specifically investigated its influence on the proliferation, apoptosis, migration, and functional differentiation of osteoblasts and osteoclasts.

**Results:**

LBP significantly promotes osteoblast proliferation, migration, and osteogenic differentiation. Conversely, it inhibits the intrinsic apoptotic response in osteoblasts. For osteoclasts, LBP suppressed their proliferation, migration, and osteoclastic differentiation while enhancing their natural apoptosis. These results were confirmed by classical protein pathway detection experiments.

**Conclusion:**

LBP showcases potential therapeutic properties against OP, particularly in modulating osteoblast/osteoclast activities. While its exact mechanisms through vital signaling pathways remain to be fully elucidated, LBP’s prominent effects suggest that it is a promising agent for OP intervention, warranting further in-depth studies.

## 1 Introduction

Osteoporosis (OP) is a group of systemic bone diseases characterized by a reduction in bone mass or, alternately, a microstructural degradation of bone tissue [1,2]. This leads to decreased bone strength, increased bone fragility, and a heightened propensity for fractures [3,4]. OP manifests as lower back pain, limb cramps, hunchbacked posture, reduced height, and even bone fractures [5,6]. Epidemiological statistics in China reveal that among the population aged above 50, the overall prevalence of OP is 20.7% for women and 14.4% for men [7]. With approximately 270 million people aged 60 and above, the figures are daunting. Moreover, international epidemiological data demonstrate that the prevalence of OP remains high in many countries. The occurrence of OP correlates with multiple factors, with gender and age having the most significant influence. Other determinants include geography, ethnicity, genetics, body mass index, and underlying health conditions [8].

OP can be broadly categorized into three primary types: primary OP (encompassing postmenopausal and senile OP), secondary OP, and idiopathic OP [9,10]. The multifaceted pathogenesis of OP primarily revolves around the fluctuations in osteoblast proliferation and apoptosis [11,12]. Apoptosis, a form of programmed cell death, is crucial for maintaining cellular homeostasis and regulating cell populations within bone tissue. It ensures the removal of damaged or excess cells, thereby preserving the delicate balance between osteoblasts and osteoclasts, which is essential for effective bone remodeling [13–17]. Critical mechanisms are orchestrated through several signaling pathways, such as OPG/RANKL/RANK, MAPK, Wnt/β-catenin, BMPs, PPAR-γ, and TGF-β [18,19]. These pathways play pivotal roles in regulating bone remodeling, bone formation, osteoblast differentiation, and osteoclast differentiation, among other processes.

*Lycium barbarum* polysaccharide (LBP), the primary effective component of the traditional Chinese medicine (TCM) Goji Berry, is composed of neutral sugars, proteins, and galacturonic acid [20]. Beyond its well-known pharmacological actions in anti-aging and immune modulation, recent research has illuminated LBP’s significant potential against OP [21]. Notably, LBP has been found to enhance bone density in rats modeled with glucocorticoid-induced OP, elevate serum calcium levels, promote alkaline phosphatase (ALP) activity, increase calcium absorption, and reduce calcium excretion [22–24]. Furthermore, LBP promotes osteoblast proliferation while inhibiting apoptosis, suggesting that its therapeutic effects against OP might be associated with modulating osteoblast activities [25,26]. The specific signaling pathways through which LBP affects osteoblasts remain to be elucidated, presenting challenges for subsequent research. Our interest in LBP stems from its unique ability to modulate cellular processes critical to bone health. Understanding these pathways could unveil novel therapeutic strategies that leverage the natural compounds in traditional medicine. This motivates our investigation into the influence of LBP on the proliferation, apoptosis, migration, and functional differentiation of osteoblasts and osteoclasts.

In this study, we explored the influence of LBP on the proliferation, apoptosis, migration, and functional differentiation of osteoblasts and osteoclasts. Our findings indicated that LBP significantly promotes osteoblast proliferation, migration, and osteogenic differentiation while inhibiting its intrinsic apoptotic response. Conversely, LBP suppressed the proliferation, migration, and osteoclastic differentiation of osteoclasts, accentuating its natural apoptosis. Further, protein-level validations elucidated and substantiated these outcomes.

## 2 Methods

### 2.1 Cell culture

MC3T3-E1 cells were purchased from the Shanghai Cell Bank of the Chinese Academy of Sciences (Shanghai, China) using α-MEM (VivaCell, Shanghai, China) complete medium, and RAW264.7 cells were purchased from the American Type Culture Collection (ATCC, USA) using Dulbecco's Modified Eagle Medium (DMEM) (VivaCell, Shanghai, China) complete medium with 10% fetal bovine serum (FBS; Gibco, USA) and 1% penicillin/streptomycin solution (VivaCell, Shanghai, China), respectively, and were placed in a CO_2_ incubator (5% CO_2_, 37°C), and were divided into groups according to the experiments [27]. LBP was added or not (Solarbio, Beijing, China).

### 2.2 Cell proliferation and migration

MTT: The cell viability of MC3T3-E1 and RAW264.7 cells after treatment with different concentrations of LBP was determined using MTT Cell Proliferation and Cytotoxicity Assay Kit (YEASEN, Shanghai, China). The cells were inoculated in 96-well plates, and when the cell fusion reached 50–60%, the cells were treated with different concentrations of LBP (0, 10, 50, 100, 250, 500, 1,000, and 2,000 μg/mL) for 24 h, and then the absorbance values were measured at 570 nm on the enzyme labeling instrument.

Wound-Healing Assay [28]: The scratch test was used to evaluate the effect of LBP on the migration ability of MC3T3-E1 and RAW264.7 cells. Cells were inoculated in six-well plates and cultured using complete medium, when the cell confluence reached 90–100%, a straight line was drawn on the bottom of the cell culture plate using a sterile pipette tip, and the cells were washed off with 1% FBS-containing medium containing 1% FBS, and then the cells were cultured in basal medium containing 1% FBS, and the control medium was not added with LBP, and the cell scratches and the area of the covered area were observed and photographed using a microscope to record the cell scratches and the area of the covered area (at 0 and 24 h).

BrdU test [29]: MC3T3-E1 cells were inoculated in six-well plates, and when the cells were 50–70% confluent, the cells were treated with medium with or without LBP for 24 h, BrdU solution was mixed into the cell culture medium and continued to be incubated for 4 h, and then subsequently fixed with 4% paraformaldehyde for 20 min at room temperature, and the subsequent operations were performed according to the BrdU test kit (Servicebio, Wuhan, China) instructions.

### 2.3 Osteogenic induction and osteoclastic induction

Osteogenic induction: MC3T3-E1 cells were inoculated in 24-well plates and cultured in α-MED complete medium until the cells were adherent to the wall, and then replaced with osteogenic induction medium [30] containing sodium β-glycerophosphate (Beyotime Biotechnology, Shanghai, China) at 10 mM, vitamin C (Beyotime) at 50 μg/mL, and dexamethasone (Beyotime Biotechnology, Shanghai, China) at 100 nM in osteogenic induction medium [30], and the medium was changed every 3 days. For ALP staining, after 7 days of osteogenic induction, the cells were fixed and stained according to the instructions of the ALP assay kit (Beyotime Biotechnology, Shanghai, China), and the proportion of positive cells was observed and counted under an inverted microscope; for alizarin red staining, after 28 days of osteogenic induction, the cells were stained using alizarin red S staining solution (Solarbio, Beijing, China), calcium salts were observed under an inverted microscope and counted.

Osteoclastic induction: RAW264.7 cells were inoculated in 24-well plates and cultured in DMEM medium until the cells were attached to the wall, and then replaced with α-MEM complete medium containing 50 ng/mL RANKL (R&D, USA) for osteoclastic induction, with the addition of LBP in the experimental group. TRAP staining kit (Servicebio, Wuhan, China) was used to identify the generation of osteoclasts, which were generally considered to be osteoclasts with ≥3 nuclei.

### 2.4 Western blot (WB)

MC3T3-E1 and RAW264.7 cells were inoculated in six-well plates, respectively. When the confluence reached 70%, complete medium with or without LBP was added, and incubated at 37°C for 24 h. After the cells were washed twice with pre-cooled phosphate buffered saline, RIPA lysate containing phenylmethanesulfonyl fluoride was added, and the cells were lysed on ice for 30 min. The cells were hung down with a spatula and collected in a centrifuge tube, centrifuged at 12,000*g* for 10 min, and the supernatant was collected as the total protein solution. These proteins were subjected to 12% SDS-PAGE and then transferred to a polyvinylidene fluoride membrane, 5% skimmed milk powder and closed at room temperature for 1 h, then incubated overnight in a 4°C refrigerator with primary antibodies: Col 1, Runx2, Osx, Ocn, Nfatc1, Cfos, Ctsk, Trap, gapdh, and tubulin (Abcam, USA or Affinity, China, 1:1,000). After incubation of horseradish peroxidase-labeled secondary antibody for 1 h at room temperature, the expression of each protein was quantitatively analyzed using Image Lab and ImageJ software [31].

### 2.5 Flow cytometry

Using Annexin V-FITC Apoptosis Detection Kit (Beyotime Biotechnology, Shanghai, China), cells were inoculated in six-well plates and different treatments were done, followed by harvesting of cells at the bottom of the dishes and in the cell culture medium according to the manufacturer’s protocols, staining and then detecting apoptosis using flow cytometry [32], and finally, analyzing the apoptosis rate using FlowJo software.

### 2.6 Statistical analysis

Data were analyzed using GraphPad Prism 9.0 and ImageJ software, error bars are mean ± SEM, significance was tested by ANOVA and unpaired *t*-test, and *P* < 0.05 was considered statistically significant. All experiments in this study were repeated a minimum of three times.

## 3 Results

### 3.1 Effect of LBP on proliferation/migration of osteoblasts and osteoclasts

**Figure 1:**
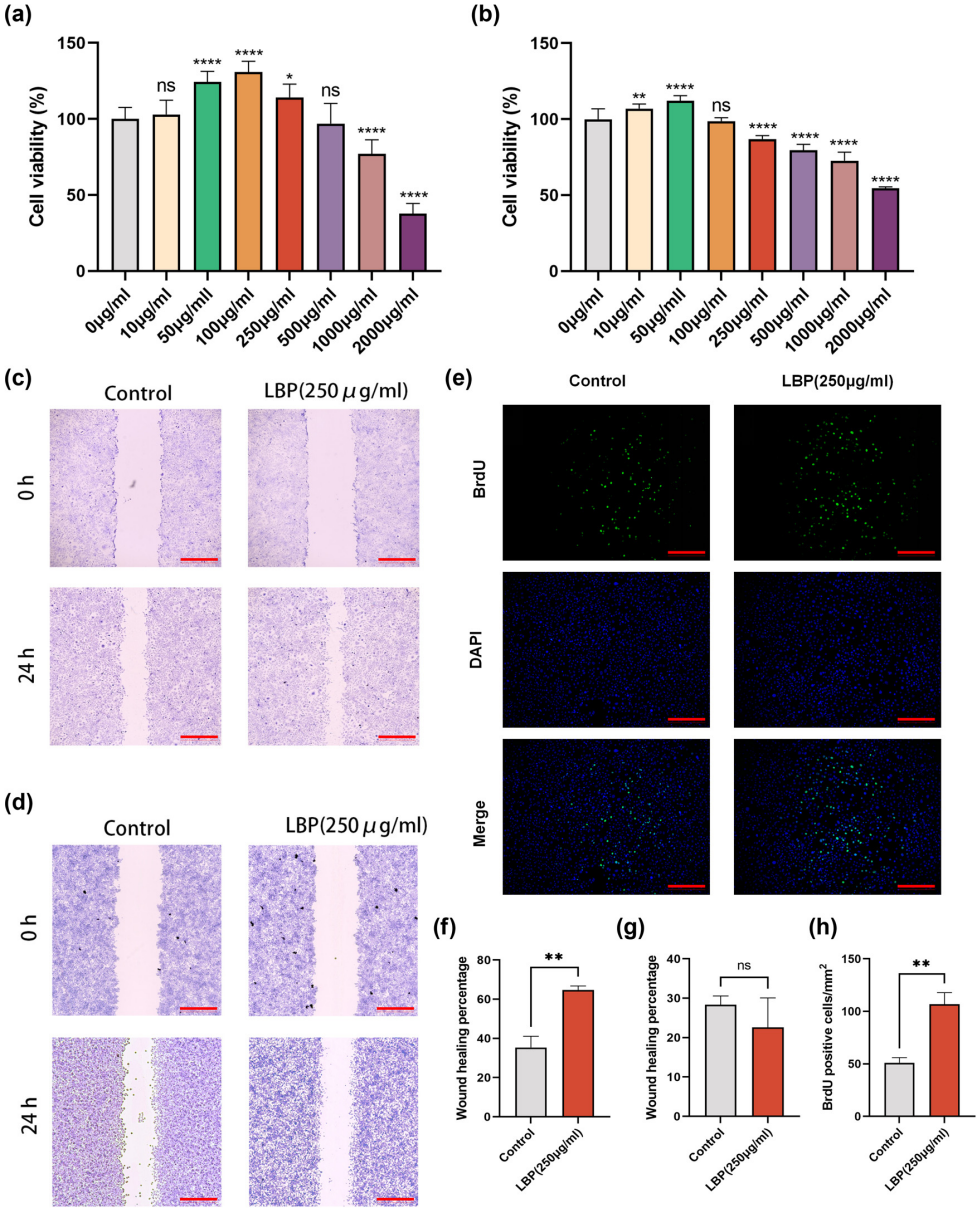
Effect of LBP on proliferation/migration of osteoblasts and osteoclasts. (a) Cell viability of MC3T3 cells after 24 h treatment with different concentrations of LBP, indicating dose-dependent responses in cellular activity. The significance of data differences between different groups was analyzed by one-way ANOVA with Bonferroni post-test, and the data were normalized. *P < 0.05, **P < 0.01, ****P < 0.0001 (c and d). (b) Assessment of cell viability in RAW cells following 24 h exposure to varying concentrations of LBP, showing alterations in metabolic activity based on LBP dosage. (c) Scratch assay conducted on MC3T3 cells demonstrating the migration and proliferation effects of LBP after a 24 h incubation period; scale bar = 200 μm. (d) Scratch assay for RAW cells to evaluate the influence of LBP on cell migration and wound healing over a 24 h duration; scale bar = 200 μm. (e) BrdU assay for MC3T3 cells demonstrating the proliferation effect of LBP after a 24 h incubation period; scale bar = 200 μm. (f)–(h) Quantitative analysis of the above results. Student’s *t*-test was used to analyze the differences between the two groups (*n* = 3). Data are presented as mean ± SD. **P < 0.01.

In order to determine the impact of different concentrations of LBP on the vitality of MC3T3-E1 and RAW264.7 cells, we designed a drug concentration gradient (0, 10, 50, 100, 250, 500, 1,000, 2,000 μg/mL), following the protocols of previous studies [33,34]. After treating the cells for 24 h, we assessed the effect of LBP on cell viability using the MTT assay. The results indicated that LBP promoted cell proliferation in a concentration-dependent manner, with lower concentrations enhancing proliferation and higher concentrations inhibiting it. Compared to RAW264.7 cells, MC3T3-E1 cells exhibited a higher threshold, with 250 μg/mL of LBP promoting proliferation of MC3T3-E1 cells while inhibiting proliferation of RAW264.7 cells (Figure 1a and b). Based on these findings, we selected 250 μg/mL LBP for subsequent experiments.

Subsequent scratch assays revealed that 250 μg/mL LBP treatment enhanced the migration ability of MC3T3-E1 cells, while no significant effect on the migration of RAW264.7 cells was observed (Figure 1c and f). However, the RAW264.7 cells density in the LBP-treated group was lower than that in the control group (Figure 1d and g). Additionally, the BrdU proliferation assay on MC3T3-E1 cells yielded similar results, with a significantly higher rate of BrdU-positive cells in the LBP group compared to the control, confirming that LBP in the culture medium promotes proliferation of MC3T3-E1 cells (Figure 1e and h).

### 3.2 LBP promotes the osteogenic differentiation of osteoblasts

**Figure 2:**
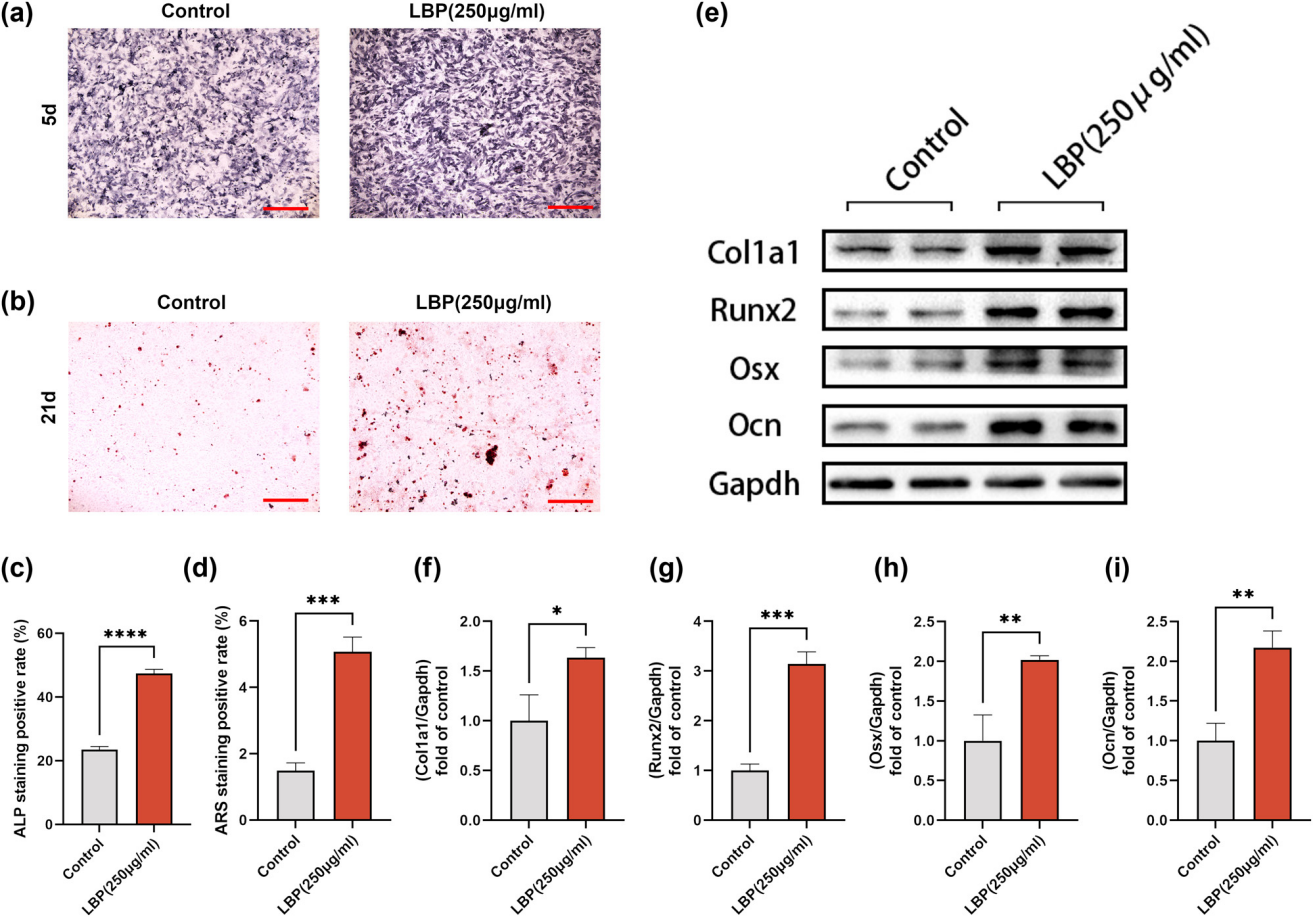
LBP promotes osteogenic differentiation of MC3T3-E1 cells. (a) ALP staining for 250 μg/mL LBP treatment (5d); scale bar = 200 μm. (b) Alizarin red staining for 250 μg/mL LBP treatment (21d); scale bar = 200 μm. (c) and (d) Quantitative analysis of the above results. Student’s *t*-test was used to analyze the differences between the two groups; ***P < 0.001, ****P < 0.0001. (e)–(i) Expression level of Col 1a1, Runx2, Osx, and Ocn in MC3T3-E1 cells was determined 24 h after different treatments by WB. GAPDH was used as an internal control (*n* = 3). Data are presented as mean ± SD. *P < 0.05, **P < 0.01, ***P < 0.001.

The osteogenic effects of LBP have been previously documented [23,35]. Here, our data corroborated these findings by showing that 250 μg/mL LBP treatment over 5 and 21 days resulted in enhanced ALP activity and increased alizarin red staining, respectively (Figure 2a–d). These outcomes are indicative of promoted osteogenic differentiation in MC3T3-E1 cells. Quantitative analyses and expression levels of osteogenesis-related genes such as Col 1a1, Runx2, Osx, and Ocn further validated the differentiation-promoting effects of LBP (Figure 2e–i).

### 3.3 LBP inhibits the osteoclastic differentiation of osteoclasts

**Figure 3:**
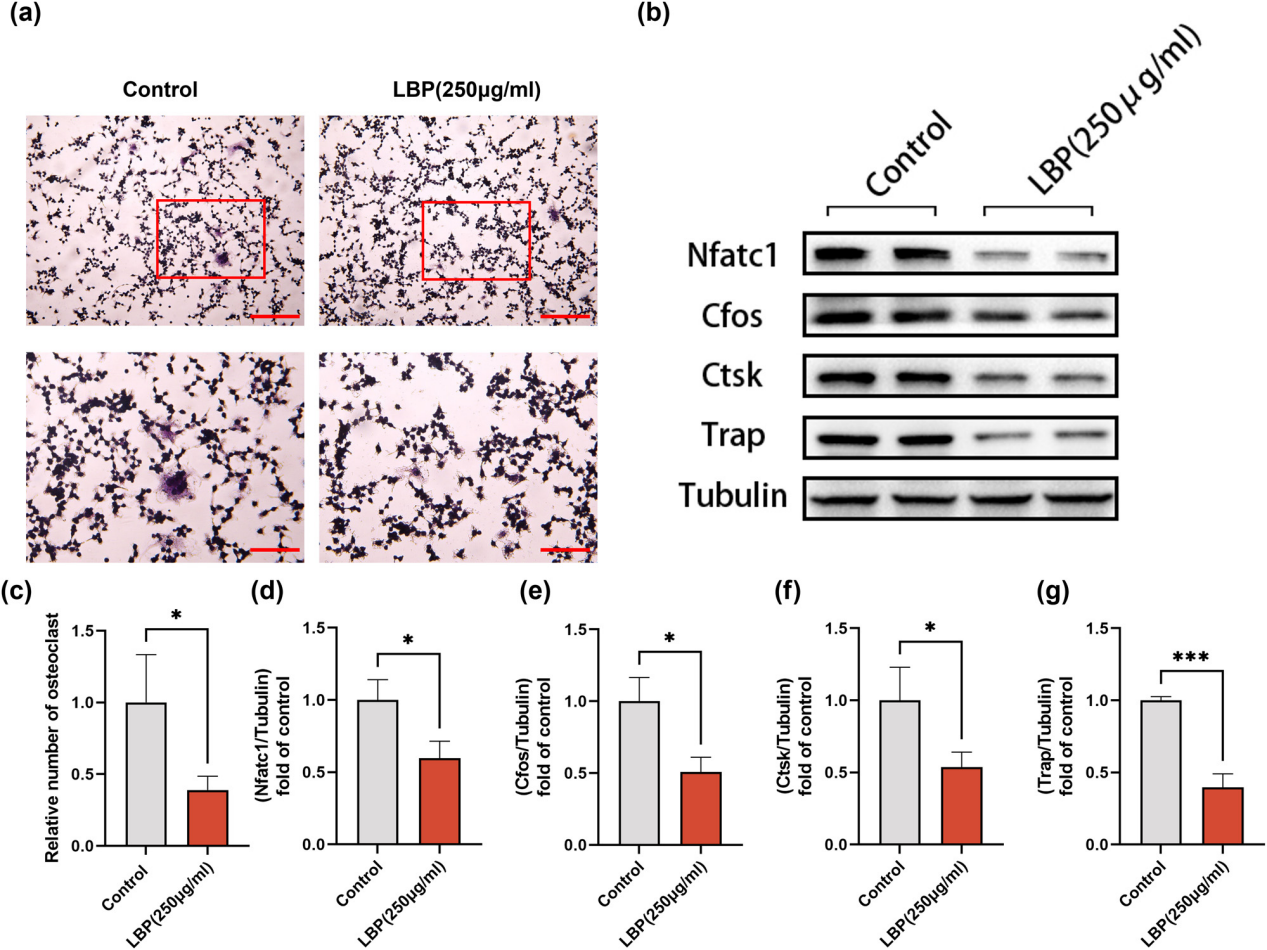
LBP inhibits osteoblastic differentiation of RAW264.7 cells. (a) TRAP staining revealed that 250 μg/mL LBP treatment inhibited RAW264.7 cells from differentiating into osteoclast; scale bar = 200/100 μm. (b) Expression levels of NFATc 1, cFOS, CTSK, and TRAP in osteoblasts *in vitro* were detected by WB. Tubulin was used as an internal control. (c)–(g) Quantitative analysis of the above results. Student’s *t*-test was used to analyze the differences between the two groups (*n* = 3). Data are presented as mean ± SD. *P < 0.05, **P < 0.01, ***P < 0.001.

The balance of osteoblastic and osteoclastic activity is crucial to bone homeostasis. Our findings suggest that LBP, at a concentration of 250 μg/mL, inhibited the osteoclastic differentiation of RAW264.7 cells as indicated by TRAP staining (Figure 3a and c). Furthermore, WB analysis showed downregulation of NFATc1, cFOS, CTSK, and TRAP expression levels in osteoblasts *in vitro* (Figure 3b and d–g). These data underscore the inhibitory effects of LBP on osteoblastic differentiation of osteoclasts.

### 3.4 LBP inhibits osteoblasts apoptosis

**Figure 4:**
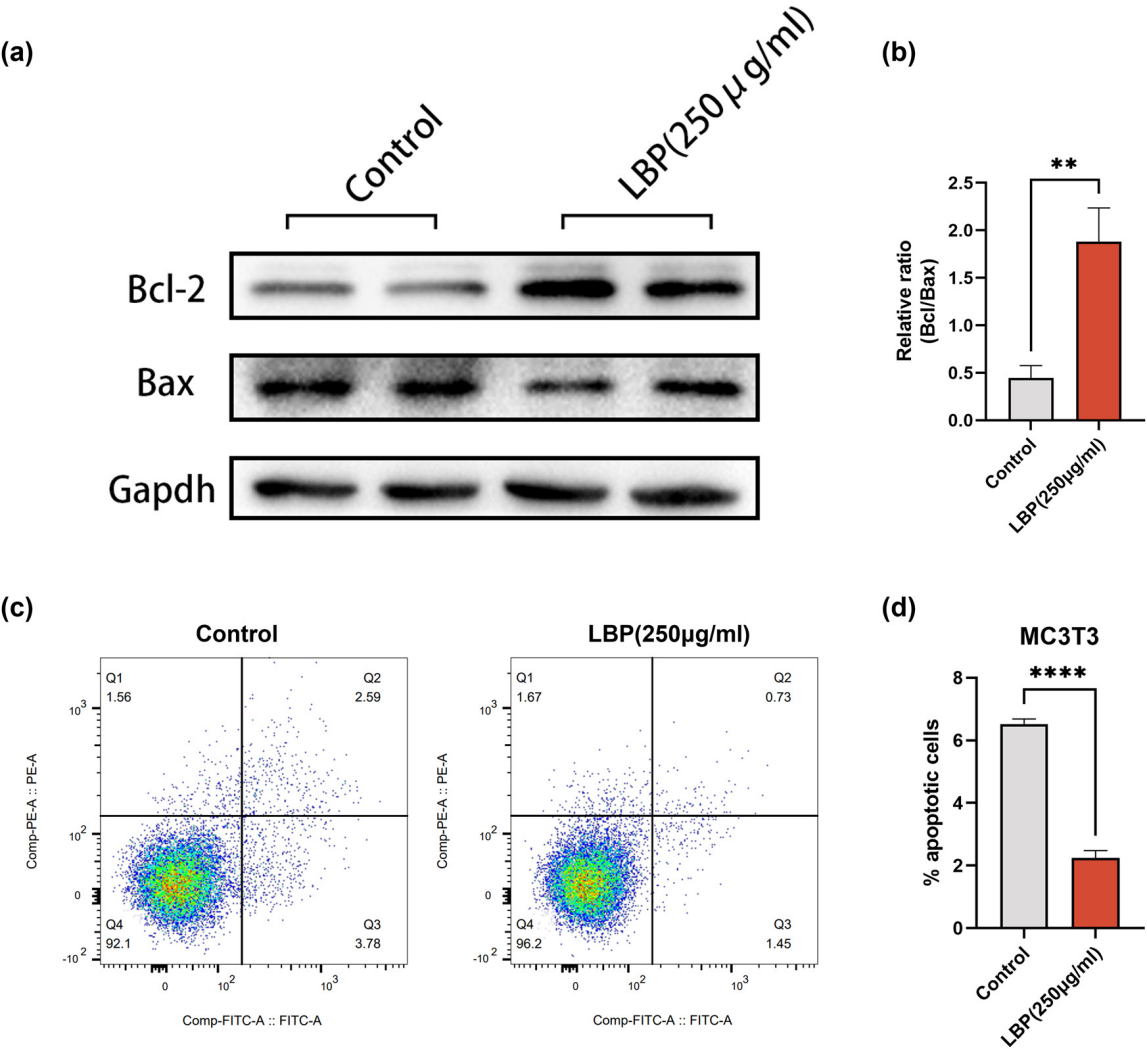
LBP inhibits osteoblast apoptosis of MC3T3 cells. (a) and (b) Expression level of Bcl-2 and Bax in MC3T3 cells was determined 24 h after 250 μg/mL LBP treatments by WB. GAPDH was used as an internal control (*n* = 3). Data are presented as mean ± SD; **P < 0.01. (c) and (d) Apoptosis flow cytometry to examine the apoptosis rate of MC3T3 before and after 250 μg/mL LBP treatment (*n* = 3). Data are presented as mean ± SD; ****P < 0.0001.

Further investigation into the anti-apoptotic effect of LBP on osteoblasts showed that 250 μg/mL LBP treatment for 24 h significantly decreased the expression levels of pro-apoptotic Bax while increasing the expression of anti-apoptotic Bcl-2 in MC3T3 cells (Figure 4a and b). Apoptosis flow cytometry confirmed a reduced apoptosis rate in MC3T3 cells after treatment with LBP (Figure 4c and d).

### 3.5 LBP enhances osteoclasts apoptosis

**Figure 5:**
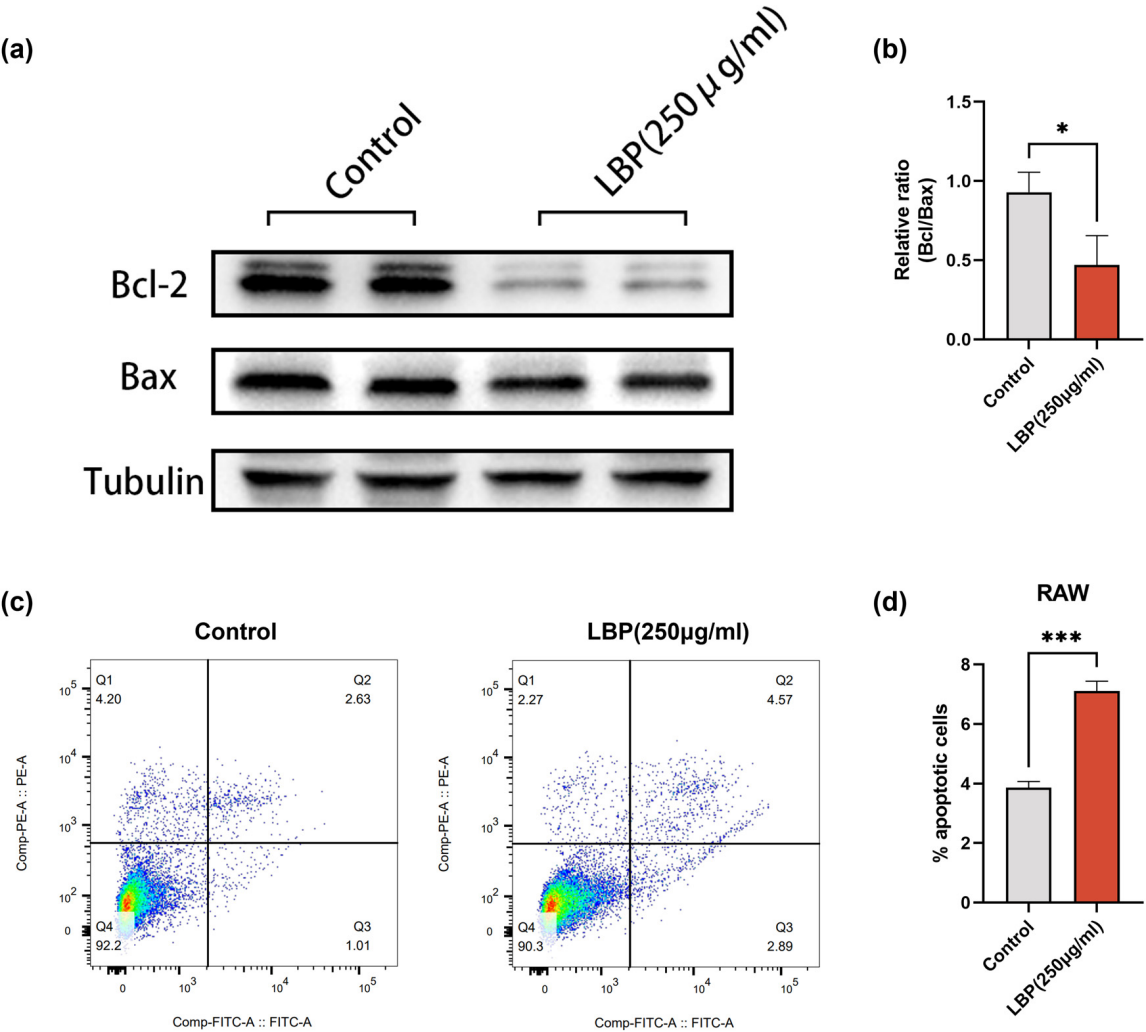
LBP promotes osteoclast apoptosis of RAW cells. (a) and (b) Expression level of Bcl-2 and Bax in RAW cells was determined 24 h after 250 μg/mL LBP treatments by WB. Tubulin was used as an internal control (*n* = 3). Data are presented as mean ± SD; *P < 0.05. (c) and (d) Apoptosis flow cytometry to examine the apoptosis rate of RAW before and after 250 μg/mL LBP treatment (*n* = 3). Data are presented as mean ± SD; ***P < 0.001.

Conversely, LBP treatment at the same concentration resulted in the opposite effect on osteoclasts. The enhanced apoptosis of RAW cells was evidenced by a considerable increased Bax/Bcl-2 ratio (Figure 5a and b). Apoptosis flow cytometry post-LBP treatment also showed an elevated apoptosis rate in RAW cells (Figure 5c and d).

**Figure 6:**
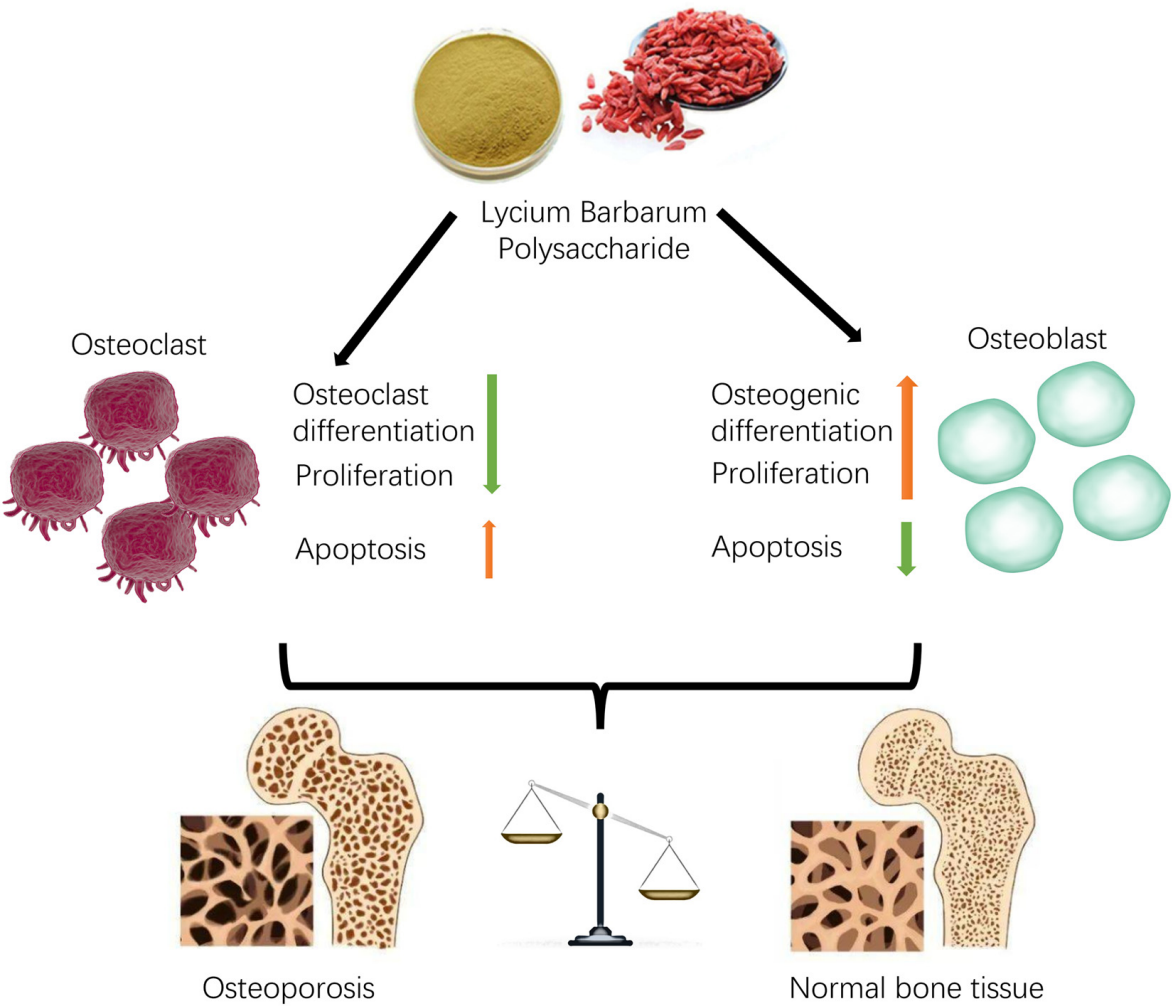
Schematic depiction of this work. LPB plays a therapeutic role in OP by modulating the proliferation, apoptosis, and differentiation capacity of osteoblasts/osteoclasts.

In conclusion, LBP may alleviate OP by alteration of osteoclasts/osteoblasts balance through apoptosis/proliferation/bone remodel (Figure 6).

## 4 Discussion

In the current study, we investigated the effects of LBP on the proliferation, apoptosis, migration, and functional differentiation of osteoblasts and osteoclasts. Our results indicate that LBP significantly promotes the proliferation, migration, and osteogenic differentiation of osteoblasts while inhibiting their natural apoptotic response. Conversely, LBP suppresses the proliferation, migration, and osteoclastic differentiation of osteoclasts, enhancing their natural apoptotic response. These findings were further validated and elucidated at the molecular protein level. In summary, this study elucidates the potential biological mechanisms by which LBP alleviates OP.

OP is categorized into three main types: primary OP, secondary OP, and idiopathic OP. Among them, postmenopausal OP and senile OP both fall under primary OP [18,36]. The pathogenesis of OP is complex, with changes in osteoblast proliferation and apoptosis being one of its crucial mechanisms. Key signaling pathways involved in osteoblast proliferation and apoptosis include OPG/RANKL/RANK, MAPK, Wnt/β-catenin, BMPs, PPAR-γ, and TGF-β′ [36–39]. The OPG/RANKL/RANK system plays a role in regulating normal bone remodeling [36]. OPG promotes bone formation, and inhibits osteoclast differentiation by blocking RANK and RANKL binding, thus suppressing bone resorption. RANKL stimulates osteoclast differentiation, leading to bone resorption [40]. The MAPK pathway primarily affects bone formation [40]. The P38 pathway promotes bone formation and suppresses bone resorption [40]. The ERK pathway plays a vital role in maintaining bone metabolic balance. The ERK5 pathway promotes the differentiation of the mononuclear-phagocyte system into osteoblasts, ensuring the expression of RANKL and OPG [41]. This pathway’s activation can reduce bone resorption and increase bone density. The Wnt/β-catenin pathway inhibits osteoclast differentiation by promoting OPG production, and its upregulation can also enhance osteoblast differentiation capabilities [41–43]. BMPs and TGF-β pathways have synergistic effects, regulating each other’s expression, and jointly controlling osteoblast proliferation and apoptosis [44,45]. Activation of the PPAR-γ pathway directs mesenchymal stem cells toward adipogenesis, playing a critical role in adipogenic differentiation, while also suppressing osteoclast differentiation through inhibiting RANKL signaling and promoting osteoblast maturation by activating osteocalcin genes [46].

Conventional OP treatments include calcium and vitamin D, hormone replacement, selective estrogen receptor modulators, and bisphosphonates [47]. Although these drugs are effective, they often have side effects, can be costly, and might not be acceptable to many patients. TCM has a long history of treating OP [45,48,49]. TCM herbs currently used for treating OP include Epimedium, Dipsacus, Cnidium, Psoralea, deer antler, Eucommia, *L. barbarum*, *Euonymus alatus*, and Drynaria [25,26]. Recent basic research and clinical trials have confirmed that some TCM herbs and natural plant extracts or their bioactive components have the potential to prevent and treat OP [50,51].

LBP is an extract from the medicinal herb *L. barbarum*, primarily composed of neutral sugars (81.37%), proteins (9.24%), and galacturonic acid (3.69%) [22]. Existing research confirms that LBP exhibits pharmacological effects in various areas such as anti-aging, immunomodulation, lipid-lowering, blood sugar reduction, hepatic and renal protection, fatigue resistance, reproductive system protection, antitumor activity, stress resistance, and hypertension prevention, showcasing its broad pharmacological activity [22,33,34]. As research deepens, recent findings have demonstrated its significant effects in intervening with OP. A study by Wang Xiaomin et al. [24] randomly divided 50 Wistar rats of both genders aged 8 weeks into five groups: control, model, high-dose LBP, low-dose LBP, and Xianling Gubao capsule groups, with ten rats in each group. An OP model was established using dexamethasone. Results indicate that LBP can increase bone density in glucocorticoid-induced osteoporotic rats, raise calcium levels in serum, enhance ALP activity, improve calcium absorption rates, and decrease calcium excretion. This suggests LBP’s potential in preventing glucocorticoid-induced OP. These studies imply that LBP possesses OP prevention capabilities, possibly linked to its regulation of osteoblast proliferation and apoptosis. However, as previously mentioned, the pathogenesis of OP is extremely intricate with numerous signaling pathways involved. The specific pathway through which LBP affects osteoblast proliferation and apoptosis to prevent OP remains undefined, representing an area of further exploration and opportunity.

In this study, our results showed that LBP significantly promotes osteoblast proliferation, migration, and osteogenic differentiation while inhibiting their natural apoptotic responses. Conversely, LBP suppresses the proliferation, migration, and osteoclastic differentiation of osteoclasts, enhancing their natural apoptotic responses. Additionally, we investigated classical pathways related to the functional differentiation of osteoblasts and osteoclasts. The results revealed that osteogenesis-related proteins such as Col1a1/Runx2/Osx/Ocn were significantly elevated post-LBP treatment in osteoblasts, suggesting LBP’s multi-dimensional, multi-pathway influence on osteogenic differentiation. For osteoclasts, LBP demonstrated significant inhibitory effects on osteoclastic differentiation-related protein molecules like Nfatc1/Cfos/Ctsk/Trap. As for the apoptotic effects on both cell types, results suggest a possible influence through the Bcl-2/Bax-related pathway. These findings align with prior observations in OP animal models, providing a comprehensive explanation for LBP’s inhibitory effects on OP.

This study possesses certain limitations: (1) we solely explored the effects of LBP on osteoblasts and osteoclasts, while other cell types such as BMSC/HUVEC are also worth examining [35]. (2) We did not conduct *in vivo* validation; thus, further studies are required for experimental verification. (3) We only investigated the most classical cellular signals for osteogenesis, osteoclastogenesis, and apoptosis [52,53]. However, the actual impact of LBP on OP may involve multiple pathways. It is crucial to understand the mechanism of LBP’s effect from a comprehensive perspective, utilizing methods like high-throughput sequencing.

## 5 Conclusion

LBP demonstrates potential therapeutic properties against OP, particularly in modulating osteoblast/osteoclast activities. While its exact mechanisms through vital signaling pathways remain to be fully elucidated, LBP’s prominent effects suggest that it is a promising agent for OP intervention, warranting further in-depth studies.
